# Drivers and consequences of partial migration in an alpine bird species

**DOI:** 10.1002/ece3.8690

**Published:** 2022-03-10

**Authors:** Øyvind Arnekleiv, Katrine Eldegard, Pål F. Moa, Lasse F. Eriksen, Erlend B. Nilsen

**Affiliations:** ^1^ Faculty of Environmental Sciences and Natural Resource Management Norwegian University of Life Sciences (NMBU) Ås Norway; ^2^ 1786 Faculty of Biosciences and Aquaculture Nord University Steinkjer Norway; ^3^ Department for Terrestrial Biodiversity Norwegian Institute for Nature Research (NINA) Trondheim Norway; ^4^ Centre for Biodiversity Dynamics (CBD) Norwegian University of Science and Technology (NTNU) Trondheim Norway

**Keywords:** alpine wildlife, eco‐evolution, *Lagopus lagopus*, migration

## Abstract

1. Partial migration, where a portion of the population migrates between winter and summer (breeding) areas and the rest remain year‐round resident, is a common phenomenon across several taxonomic groups. Several hypotheses have been put forward to explain why some individuals migrate while others stay resident, as well as the fitness consequences of the different strategies. Yet, the drivers and consequences of the decision to migrate or not are poorly understood.
2. We used data from radio‐tagged female (*n* = 73) willow ptarmigan *Lagopus lagopus* in an alpine study area in Central Norway to test if (i) the decision to migrate was dependent on individual state variables (age and body weight), (ii) individuals repeated migratory decisions between seasons, and (iii) the choice of migratory strategy was related to reproductive success.
3. Partially supporting our prediction that migratory strategy depends on individual state, we found that juvenile birds with small body sizes were more likely to migrate, whereas large juveniles remained resident. For adult females, we found no relationship between the decision to migrate or stay resident and body weight. We found evidence for high individual repeatability of migratory decision between seasons. Migratory strategy did not explain variation in clutch size or nest fate among individuals, suggesting no direct influence of the chosen strategy on reproductive success.
4. Our results indicate that partial migration in willow ptarmigan is related to juvenile body weight, and that migratory behavior becomes a part of the individual life history as a fixed strategy. Nesting success was not affected by migratory strategy in our study population, but future studies should assess other traits to further test potential fitness consequences.

## Graphical abstract

We investigated causes and consequences of partial migration in Willow Ptarmigan. We found that body mass affected propensity to migrate in young birds but not among older birds, and that migration was a highly repeatable trait.

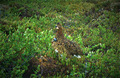

## 1 INTRODUCTION

Migration between distinct breeding and wintering areas is a widespread behavioral trait in many species across a wide range of taxa, and is generally assumed to be an adaptation to seasonal variation in environmental conditions (Reid et al., 2018). Such seasonal migrations can increase individual fitness (Alerstam et al., 2003; Somveille et al., 2015), as it allows the birds to utilize highly productive habitats all year‐round. In contrast, other bird species do not perform long‐distance seasonal migrations, as they are adapted to remain at high latitudes throughout the entire year and survive the low‐productive winters (Barta et al., 2006; Svorkmo‐Lundberg et al., 2006). However, species that display such behavior may perform shorter migrations between summer and winter areas in heterogeneous landscapes where availability and/or quality of resources vary between seasons (Barraquand & Benhamou, 2008; Fedy et al., 2012). Some overwintering populations are partially migratory (Chapman et al., 2011), implying that a portion of the population migrates between summer and winter areas, whereas the rest stay resident.

Partial migration has received considerable attention in the literature in the last decade (Berg et al., 2019; Chapman et al., 2011; Cobben & van Noordwijk, 2017; Hegemann et al., 2019; Pulido, 2011; Reid et al., 2018), and several hypotheses have been put forward to explain both within‐species and within‐population variation in migratory behavior. Lundberg (1987, 1988) suggested that the evolution of partial migration could be explained by two alternative hypotheses. First, it could evolve (i) as a frequency‐dependent evolutionary stable strategy (ESS) with two phenotypic tactics – or genetic dimorphism with two coexisting morphs (i.e., migrants and residents) – with equal fitness payoffs. Second, partial migration could evolve (ii) as a conditional strategy where individual state variables and interactions with environmental factors determine the decision to migrate or not at the individual level. Moreover, three well‐established hypotheses have been put forward to explain the drivers behind partial migration based on individual traits (i.e., conditional strategies; Chapman et al., 2011). These traits can be individual fixed‐state variables such as age and sex, or plastic state variables such as body condition (Lundberg, 1988). The body size hypotheses (Hegemann et al., 2015; Ketterson & Nolan, 1976) suggest that large individuals are more likely to stay resident due to higher ability to endure seasonal fluctuations in food abundance and temperature/weather conditions, whereas smaller individuals are more likely to migrate to habitats with more benign environmental conditions. In the traditional form, the body size hypothesis states that large body mass is most advantageous during winter due to higher thermal or nutritious stress in this season (Chapman et al., 2011; but see Alonso et al., 2009). The dominance hypotheses (Gauthreaux, 1982) suggest that dominant (often larger) individuals have a competitive advantage in environments with limited food resources (Mysterud et al., 2011) or nesting sites (Gillis et al., 2008), which could trigger migration in smaller or sub‐dominant individuals. The arrival time hypothesis (Ketterson & Nolan, 1976) suggests that because of earlier nest site occupancy and higher fitness of early arriving birds, individuals arriving early at the breeding site have higher reproductive success. Hence, birds that stay in the territory year‐round, are expected to have higher reproductive success. In cases where there is intrasexual competition for breeding sites, some individuals might decide to migrate. The body size, dominance, and arrival time hypotheses suggest that the decision to migrate or stay in the area year‐round is influenced by individual state, intraspecific interactions, or environmental conditions, and that the fitness reward from the two alternative strategies can differ. These different hypotheses might play out differently in populations where residents and migrants share a non‐breeding habitat but breed allopatrically (i.e., *breeding partial migration*) and in populations where residents and migrants share a breeding habitat but live allopatrically during the non‐breeding season (i.e., *non*‐*breeding partial migration*; Chapman et al., 2011). So far, most research has focused on non‐breeding partial migration, but breeding partial migration has been studied in, e.g., American dippers *Cinclus mexicanus* (Gillis et al., 2008).

The fitness consequences of being resident vs. migratory in a partially migratory population are poorly understood (Berg et al., 2019; Chapman et al., 2011). Nevertheless, differences between resident and migratory individuals in fitness parameters such as survival and reproduction have been suggested in theoretical and reported from empirical studies. Theoretical studies suggest that a conditional strategy can result in unequal fitness between strategies in partially migratory populations (Chapman et al., 2011; Kokko, 2011; Lundberg, 1987, 1988). Most empirical studies also report fitness to differ between migratory strategies (Buchan et al., 2019). For instance, in a partially migratory population of American dippers, Gillis et al. (2008) found that migrants had lower reproductive success but higher survival rates compared to resident individuals. The higher survival rates did, however, not offset the lower reproductivity. Adriaensen & Dhondt (1990) found both higher survival and reproductive success in resident European robins *Erithacus rubecula* and hypothesized that the differences could be attributed to a conditional strategy. In contrast, Hegemann et al. (2015) found no differences in reproductive success between migrants and residents in a skylark *Alauda arvensis* population, despite higher average body mass in resident birds. Both theoretical and empirical studies generally suggest migration to be a losing strategy within partially migrating populations, and that the decision to migrate may be to make “the best of a bad job” (Chapman et al., 2011).

**FIGURE 1:**
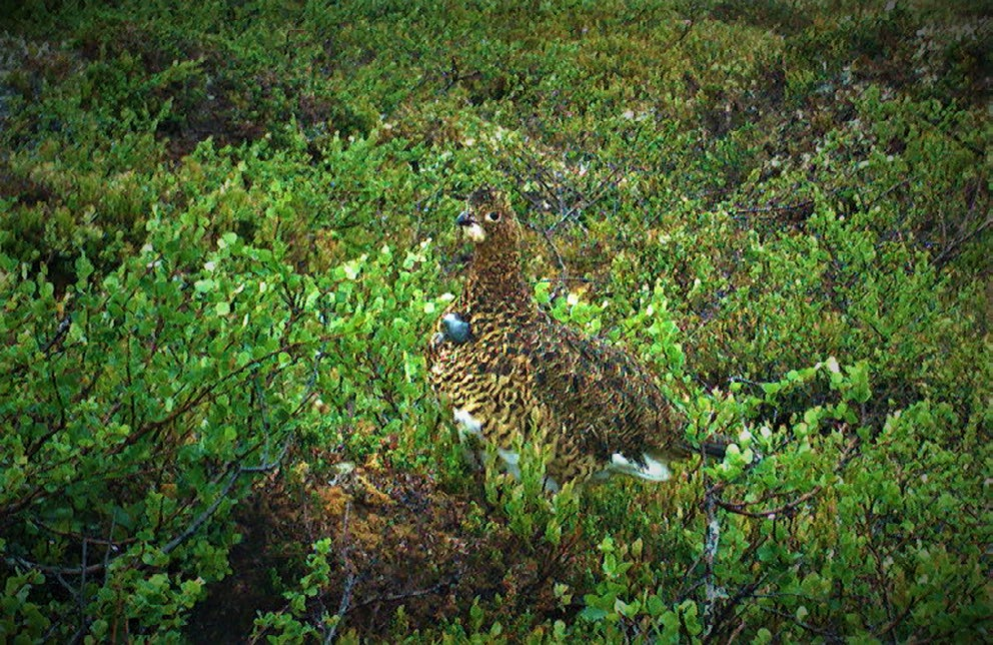
Radio marked willow ptarmigan female. Photo is taken by an automatic game camera mounted at the females nest

Empirical studies on potential fitness consequences of partial migration have so far been limited to passerines, although partial migration is a common phenomenon reported in multiple bird orders, including Galliformes (Cade & Hoffman, 1993; Chapman et al., 2011; Grist et al., 2017; Holte et al., 2016). The willow ptarmigan *Lagopus lagopus* (Figure 1) is a tetraonid bird with a circumpolar distribution (Fuglei et al., 2020), which lives year‐round in heterogeneous alpine and artic ecosystems. Because male willow ptarmigans regularly display polygamy, male breeding success is therefore more difficult to quantify than female breeding success and consequently more often unknown (Tarasov, 2003). Several studies have reported migratory behavior in ptarmigan populations (Brøseth et al., 2005; Gruys, 1993; Hoffman & Braun, 1975; Hörnell‐Willebrand et al., 2014; Irving et al., 1967; Nilsen et al., 2020). From Sweden, Hörnell‐Willebrand et al. (2014) reported considerable individual variation in seasonal migration distances in willow ptarmigan, with some individuals considered to be residents and others to be migrants. Empirical data from other Scandinavian ptarmigan populations imply non‐migratory behavior (Pedersen et al., 2003), suggesting that there are both inter‐ and intrapopulation differences in the propensity to migrate between summer and winter areas in willow ptarmigan. Willow ptarmigans from some populations often gather in distinct wintering areas (Weeden, 1964), which suggests these populations to be breeding partially migratory (Chapman et al., 2011) due to some individuals migrating to breeding areas during spring while others stay resident, either in the wintering or in the breeding areas. Currently, the drivers and consequences of partial migration in willow ptarmigan are poorly understood.

Here, we test a number of predictions from a preregistered hypothesis (Arnekleiv et al., 2019; Nilsen et al., 2020) put forward to explain causes and consequences of partial migration behavior in female willow ptarmigan. We focused on females only because we did not have access to reproductive success data from males in our study population. Assuming that migrants are making the best of a bad job (Lundberg, 1987), and based on the hypotheses about state‐dependent evolution of partial migration in birds outlined above, we predict that:

1. Female willow ptarmigans with (a) large body size are more likely to remain resident than females with smaller body size, and (b) juveniles are more likely to be migrants than adults.
2. Migration is not a fixed strategy in female willow ptarmigan.
3. Resident female willow ptarmigans have higher nesting success than migrants.

Under the assumption that winter is the most thermally or energetically constraining season as implied in the traditional form of the body size hypothesis (Chapman et al., 2011; Ketterson & Nolan, 1976), our data would not allow for an efficient test of this hypothesis. The body size hypothesis would typically be tested with data from systems with non‐breeding partial migration, as defined above. The predictions were preregistered (Nilsen, Bowler, et al., 2020) at the Open Science Framework (OSF) prior to analyzing data (Arnekleiv et al., 2019).

## 2 METHODS

### 2.1 Study area

**FIGURE 2:**
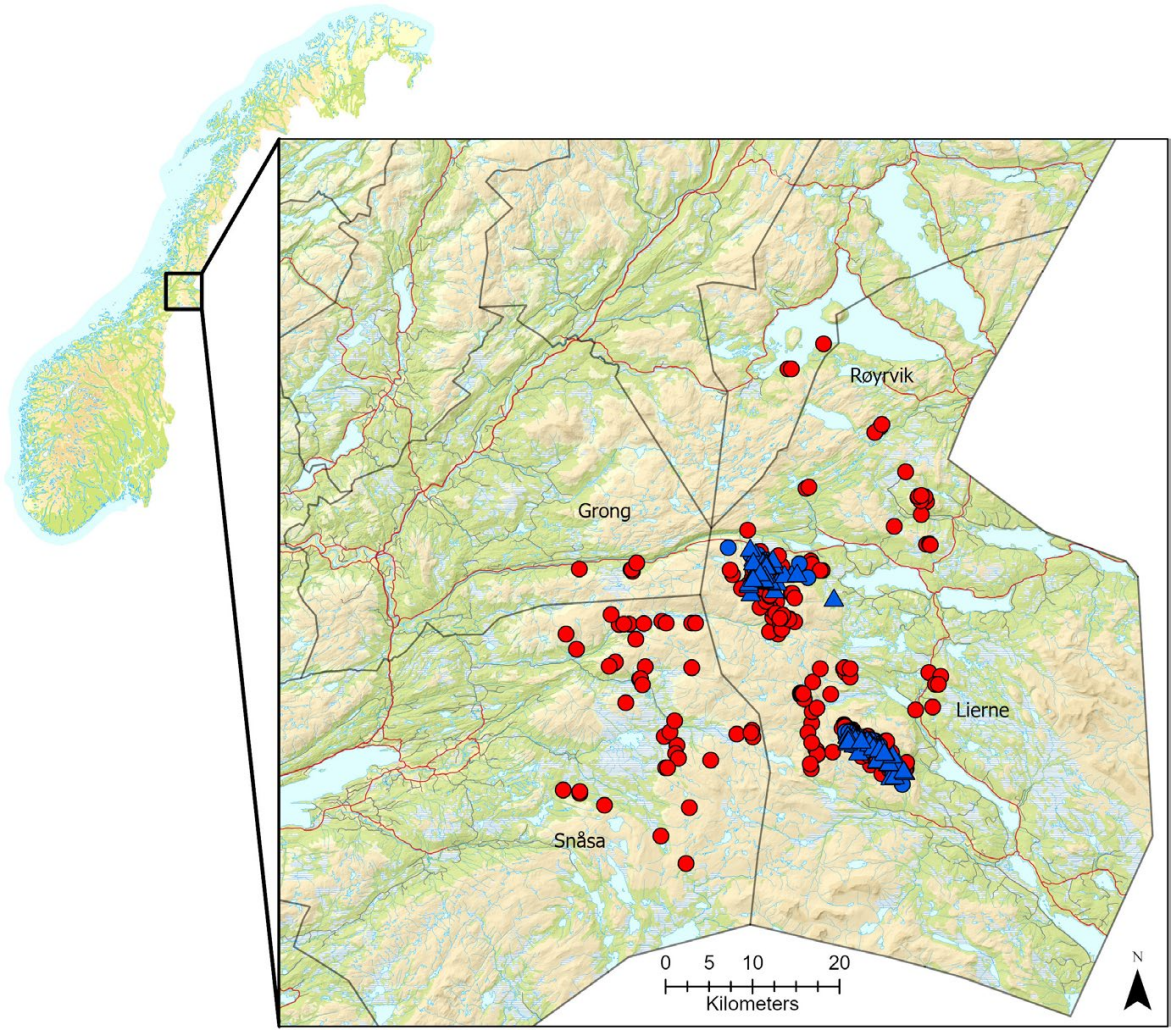
Triangulated positions of all female willow ptarmigan during the study period in the winter (January–March, blue circles) and summer (May–July, red circles) seasons. The blue triangles represent capture locations; the northern cluster is Lifjellet capture site and the southern cluster is Guslia capture site. Map to the left shows the location of the study area in Central Norway

The study was conducted in Lierne municipality in the northeastern part of Trøndelag County, Norway, with minor extensions of the study area into neighboring municipalities Snåsa, Røyrvik, and Grong due to longer movements from the main study area by some individuals (Figure 1). Ptarmigans were captured at two sites (Guslia and Lifjellet), which were located 20 km apart near Blåfjella‐Skjækerfjella National Park (Figure 2). Both in winter and summer, willow ptarmigans are distributed across the larger study area, and some birds overwinter also in the breeding areas of the migratory birds from this study. Because we only captured birds during winter at two specific capture areas, birds that were resident at other sites in the larger study area would not be available for capture in our study. This also limited our ability to test the body size hypothesis. The study area was situated in the low alpine and north boreal bioclimatic zones (Moen, 1999); the low alpine zone was dominated by *Salix* spp., dwarf birch *Betula nana*, and *Ericaceae* spp. interspersed with birch *Betula pubescens*, whereas the north boreal zone was dominated by Norway spruce *Picea abies*, Scots pine *Pinus sylvestris*, birch *Betula* spp., Ericaceae dwarf shrubs, and bryophytes.

### 2.2 Field data collection

Willow ptarmigans were captured during February and March during winter 2015–2019. The birds were spotted from snowmobiles during night‐time and temporarily blinded with powerful headlamps and caught with long‐handled dip‐nets (Brøseth et al., 2005; Hörnell‐Willebrand et al., 2014; Sandercock et al., 2011). Body weight (measured with Pesola LightLine 1000 g spring scale – rounded to nearest 5 g) and wing length (measured with Axminster Workshop Hook Rule 300 mm – carpal to tip of longest primary of flattened wing, measured to nearest mm) were measured prior to instrumenting the birds with radio collars. Captured birds were identified in the field as either female or male based on saturation of red in the eyebrow, where males have more pronounced red color than females (Pedersen & Karlsen, 2007). One feather was collected for DNA analyses to confirm sex, and the genetic marker Z‐054 (Dawson et al., 2015) was used to determine the sex of the bird. Eighty‐five percent of the sex assignments in the field were correct (Israelsen et al., 2020). Captured birds were also classified into juvenile (captured during the first winter following the year of birth) and adult (2nd year +) based on the amount of pigments in primary feathers 8 and 9, where juveniles have more black pigments in 9 than in 8 (Bergerud et al., 1963). Each individual was marked with a stainless steel ring with a unique identification number. Most of the birds were equipped with a VHF radio tag (Holohil – RI‐2DM, 14.1 g) on the 152 MHz frequency band. For all marked birds, the combined weight of the leg ring and radio transmitter was <3.5% of the body weight. Radio transmitters were programmed to send mortality signals after recording no movement for more than 12 h. In March 2018, five ptarmigans were captured and marked with GPS transmitters (Milsar – GsmRadioTag‐S9, 12 g). The transmitters sent position data over the GSM network every 4th hour.

Willow ptarmigan positions were for the most part collected once a month by manual tracking on foot by triangulation, using handheld receivers (Followit – RX98) and antennas (Followit – four‐element Yagi‐antenna); 2–5 bearings were used to determine best position and the distance between each telemetry location varied from 0.3 to 1 km. If only two bearings were obtained, the cross‐section was included when the terrain indicated that the observation was trustworthy (e.g., when the cross‐bearing pointed to a position in the end of a valley). Few positions were collected in January and December due to short day length and challenging weather conditions. To avoid loss of data due to long‐distance movements, we conducted wider aerial triangulation using a helicopter or fixed‐winged airplane three times a year (May, September, and November) in the years 2016–2019. In 2015, we only conducted triangulation from the air in October. Additional positions were either on‐site direct observations from captures or homing in on individuals.

Nesting success in spring was first assessed by homing in on radio‐tagged females to check whether they were nesting. Furthermore, incubating females were flushed off the nest, eggs were counted, and a wildlife camera (Reconyx HF2X Hyperfire 2 or Wingcam II TL) with movement sensor was deployed 2–5 m from each nest. The nests were revisited in July after hatching to determine the fate of the nest by inspecting and counting the eggshells to see whether and how many eggs were hatched or predated. In addition, pictures from the cameras were examined.

### 2.3 Classification of migratory behavior

**FIGURE 3:**
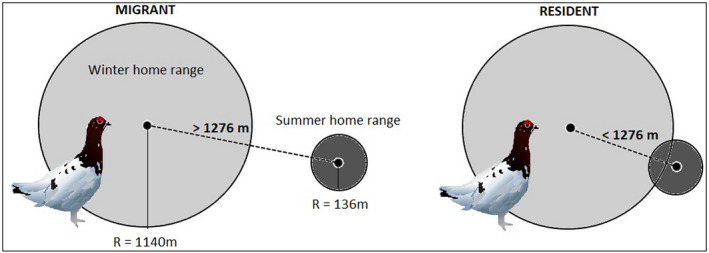
Female ptarmigans were classified as either migrants, if the distance between the activity center of winter and summer home ranges exceeded 1276 m (i.e., no overlap), or residents, if the distance between the centroids of winter and summer home range was less than 1276 m (i.e., overlap)

To examine migratory movements between seasons, we classified January–March as winter and May–July as summer. Of a total of *n* = 101 captured female ptarmigans, only females with data from at least one winter and the consecutive summer season were included in the analysis (*n* = 73) (Table 1). We collected 1–2 positions per individual in the winter and 1–5 positions per individual during summer. For each female in each season, migratory decisions were determined based on whether or not there was overlap between the winter home range and the consecutive summer home range (Figure 3), and between the summer home range and the consecutive winter home range.

**TABLE 1:** Number of radio‐tagged female willow ptarmigan captured in the capture sites Guslia and Lifjellet. *N* observations/nests show the total number of individual migratory decisions and nests included in the analysis of the first spring transitions from winter to summer areas. The numbers in parentheses show number of observations/nests when repeated decisions for some birds, and both spring and autumn movements, were included in the mixed effects models presented in Appendix S1

Year	Guslia	Lifjellet	*N* marked	*N* observations included in analyses	*N* nests included in analyses
2015	14	6	20	14 (14)	10 (10)
2016	10	10	20	16 (23)	13 (14)
2017	8	12	20	14 (24)	6 (7)
2018	4	13	17	11 (20)	11 (13)
2019	11	13	24	18 (23)	16 (18)
Total	47	54	101	73 (104)	56 (62)

Due to the limited amount of location data for each individual, we were not able to use the more data hungry approaches that have been developed for research on GPS‐tagged individuals (Cagnacci et al., 2016). Thus, we opted to create a decision rule for classification of migratory decision based on the available data and the assumption that all females shared a common home range size in summer and winter, respectively. We used the following approach:

First, we calculated an average winter home range size from positions of three of the GPS‐tagged ptarmigan during the winter 2018, all marked in March 2018. Individual home range sizes were calculated as 95% Minimum Convex Polygons (MCP) using the function *mcp* in R package adehabitatHR (Calenge, 2006). The average 95% MCP for the three GPS‐tagged ptarmigans was 4.08 km^2^. Before calculating the individual 95% MCPs, we removed inaccurate positions (due to GPS error). We defined a position as an outlier if the distance between two consecutive positions (i.e., time *t* and *t *− 1, respectively) was more than two times the distance between positions surrounding the focal position (i.e., distance between position taken at *t *− 1 and *t* + 1). Positions from the GPS‐tagged ptarmigan were only used to estimate the average “baseline” winter home range size, and these birds were not included in further analyses. For each of the VHF‐tagged females included in the analyses, we assumed that they had a circular winter home range equal to the size calculated from the GPS data (4.08 km^2^ (radius = 1140 m)) centered around the activity center (determined by triangulation) of each female in each winter season; this was used as a proxy for individual winter home range size and location.

Second, we estimated the size of the summer home ranges using data from VHF‐tagged female ptarmigan with ≥3 positions during the summer season (May–July). For each female, we drew a polygon based on the positions, and calculated the area of the polygon. As a measure of a “baseline” summer home range for further analysis, we used the median of all the individual summer home range sizes (*n* = 46). The baseline home range area was estimated to be 0.058 km^2^, corresponding to a circular home range with radius = 136 m. This size is in good agreement with previous studies of ptarmigan summer home range sizes (Eason & Hannon, 2003). For each of the females included in the analyses, we assumed a circular summer home range of 0.058 km^2^ (radius = 136 m) centered around the activity center (determined by triangulation and nest location) of each female in each summer season, as a proxy for individual summer home range. When calculating the activity center, the activity center for nesting hens (*n* = 68) was shifted toward the nest location, by assigning equal weights to the position of the nest and the sum of all other positions. All spatial computations were done using R (R Core Team, 2019).

Females with overlapping winter/summer or summer/winter home ranges were classified as residents, whereas females with no overlap were classified as migrants. Based on the “baseline” home range sizes, ptarmigans moving further than 1276 m (radius winter home range + radius summer home range) were consequently classified as migrants and females moving less than 1276 m were classified as residents.

### 2.4 Statistical analysis

To test our predictions about state‐dependent migration strategy, we used generalized linear models (GLM) based on data from the first spring migratory decision for each bird. Although this limited our sample size, it allowed a more stringent test of the migratory decisions from a sympatric wintering area to allopatric breeding area (i.e., *breeding partial migration*). Migratory decision was modeled as a binary response variable (see above), and body weight, age, and body weight × age interaction as fixed explanatory terms. Body weight was used as a measure of body size. Body weight can, however, fluctuate across short and long time intervals, and such intraindividual variation might make body weight a less reliable measure of body size; we acknowledge this limitation of the current study. For all models, the body weight variable was standardized by extracting the mean and dividing by the standard deviation. Under the assumption that migratory decisions are (relatively) fixed and symmetrical across seasons, we also analyzed the data using generalized linear mixed effects models including all observations (*glmmTMB* function in R package glmmTMB; Brooks et al., 2017), with migratory decision as a binary response variable and bird identity included as random effect to account for repeated observations of individual birds. Note that this approach included both spring and autumn migration decisions. The results from the mixed effects models are presented in Appendix S1.

As an additional test of prediction 1, we also tested whether the distance migrated was influenced by age and body weight by fitting linear models (GLM) with log(movement distance) as response variable, and weight, age, and the weight × age interaction as fixed explanatory terms. We used an identity link function (assuming a Gaussian distribution of the residuals), and included only the first spring migratory decision for each bird. As above, we repeated the analyses including all data (i.e., repeated observations for some birds, and including both spring and autumn migratory decisions), we used generalized linear mixed effects models (*glmmTMB* function in R package glmmTMB), including bird identity as intercept term to account for repeated observations of individual birds.

To assess if the decision to migrate or not was a fixed strategy in female willow ptarmigan, we estimated the repeatability $R_{M}$ in a mixed‐effect model with log(movement distance) as response variable. Only females with two or more observations of seasonal migration decisions were included. We also assessed models for repeatability in migratory decision (binary response), but do not report those due to convergence failure. Repeatability $R_{M}$ was estimated as the proportion of the total variance that was attributed to within‐group (bird identity) variation (Sokal & Rohlf, 1995):

$$R_{M}=\frac{{\sigma \;}_{\alpha }^{2}}{\sigma_{\alpha }^{2}\;+\sigma_{\varepsilon }^{2}}$$

Agreement repeatability was estimated based on the intercept‐only model (i.e., not accounting for any fixed factors), whereas adjusted repeatability was estimated with age included as a fixed‐effect term in the model (Nakagawa & Schielzeth, 2010). Repeatability was calculated using the rptR package (Stoffel et al., 2017), and the 95% confidence interval for the repeatability was estimated using parametric bootstrapping (*n* = 1000).

To test whether reproductive success was influenced by migratory strategy, we 1) fitted generalized linear models with number of eggs as response variable; migratory decision, age, weight, and year as explanatory variables; and bird identity as random effect. Because clutch size data are often underdispersed (Kendall & Wittmann 2010), we used a Conway–Maxwell–Poisson distribution that includes an additional parameter (ϕ) that accounts for violations in the mean‐variance assumption in a standard Poisson distribution. The models were fitted to the data from the first spring after capture for each bird using the function *glm*.*cmp* in package mpcmp (Fung et al., 2020). Then, (2) we fitted generalized linear model with nest fate as binary response variable (i.e., hatched chicks vs. predated or abandoned nest) and migratory decisions, age, weight, and year as explanatory variables and with bird identity as random effect. We repeated the analyses including all observations (i.e., more than 1 year for some birds) using generalized linear mixed effects models (*glmmTMB* function in R package glmmTMB). The results from the mixed effects models are presented in Appendix S1.

All model selection was based on the Akaike's information criterion corrected for small sample sizes (AIC_c_) (see, e.g., Bolker et al., 2008). The AIC_c_ encourages parsimony by adding a term to penalize more complex (larger number of parameters) models (e.g., Bolker et al., 2008).

Data and R‐code are available from an open archive hosted by the Open Science Framework (Arnekleiv et al., 2022).

## 3 RESULTS

### 3.1 Migration strategy in relation to age and body weight

**FIGURE 4:**
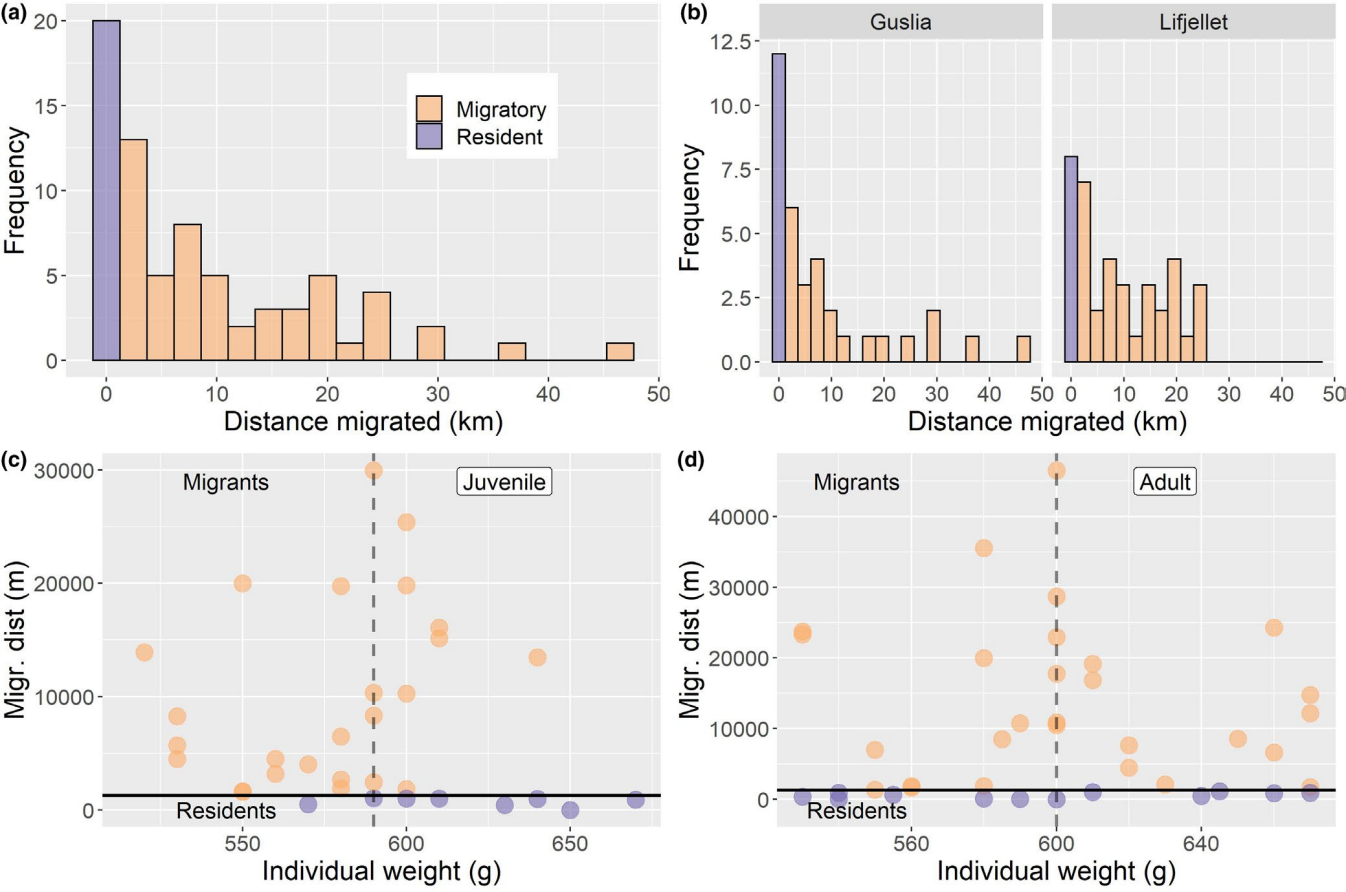
(a) Distribution of seasonal migration distances for female willow ptarmigan. Purple bar represents resident individuals, and orange bars represent migrants. See Figure 2 for definition of resident and migratory individuals. (b) Migration distance plotted for each capture site. (c) Distances migrated plotted against body weights of individual juvenile birds. Dashed vertical line represents mean and median weight and solid horizontal line marks the threshold movement distance separating residents and migrants (1276 m). (d) Same as c, but for adult birds. Purple dots represent migrants, whereas orange dots represent residents. In all panels, only winter‐to‐summer transitions are included, and only first year of data for each bird

A total of 104 cases of seasonal movement behaviors (i.e., decisions to migrate or remain resident) were included in this study (Table 2), of which 87 were winter area to summer area movements and 17 were movements from the summer area to the winter area. When including only transitions from winter to summer areas, three times as many cases of migratory (*n* = 53, 73%) than of resident (*n* = 20, 27%) behaviors were observed (Table 2). Mean and median movement distances – for both juvenile and adult females – were substantially longer than the distance limit for being classified as migrant (1276 m; Table 3). Overall, 67% of the seasonal movement distances were shorter than 10 km, 25% were between 10 and 25 km, whereas only a few (8%) seasonal movements were longer than 25 km (Figure 4). In general, seasonal movement distances were longer for birds marked at Guslia compared to birds marked at Lifjellet (Figure 3). Mean and median differences in weight between juveniles and adults were small (Table 3). There was no evidence for a difference (*p* = .70 – linear model) in elevation of the nest site locations between residents (mean elevation: 593 m.a.s. ±23) and migrants (583 m.a.s. ±16).

**TABLE 2:** Distribution of decisions to migrate or remain resident from winter to summer (first year of data after capture only) observed for 73 female willow ptarmigans during the 5‐year study period. The numbers in parentheses include all observations of migratory decisions, both from winter to consecutive summer and from summer to consecutive winter

Year	Residents	Migrants	Total	% Migrants
2015	6 (6)	8 (8)	14 (14)	57 (57)
2016	5 (5)	11 (18)	16 (23)	69 (78)
2017	5 (5)	9 (19)	14 (24)	64 (79)
2018	1 (4)	10 (16)	11 (20)	91 (80)
2019	3 (6)	15 (17)	18 (23)	83 (74)
Total	20 (26)	53 (78)	73 (104)	73 (75)

**TABLE 3:** Distance moved from winter to summer area (first year of data after capture only) and weight of juvenile and adult female willow ptarmigans. N is the total number of movement distances observed. For adults, the numbers in parentheses include all observations, both from winter to consecutive summer and from summer to consecutive winter. Weight data are from capture during winter (March), rounded to nearest 5 g

	Age	Min.	Mean	Median	Max.	*N*
Distance (km)	Juv	0.0	7.8	4.5	30.0	33
	Ad	0.0	9.9 (9.6)	6.8 (7.0)	46.5 (46.5)	40 (71)
Weight (g)	Juv	520	590	590	670	33
	Ad	530	600	600	670	40

**FIGURE 5:**
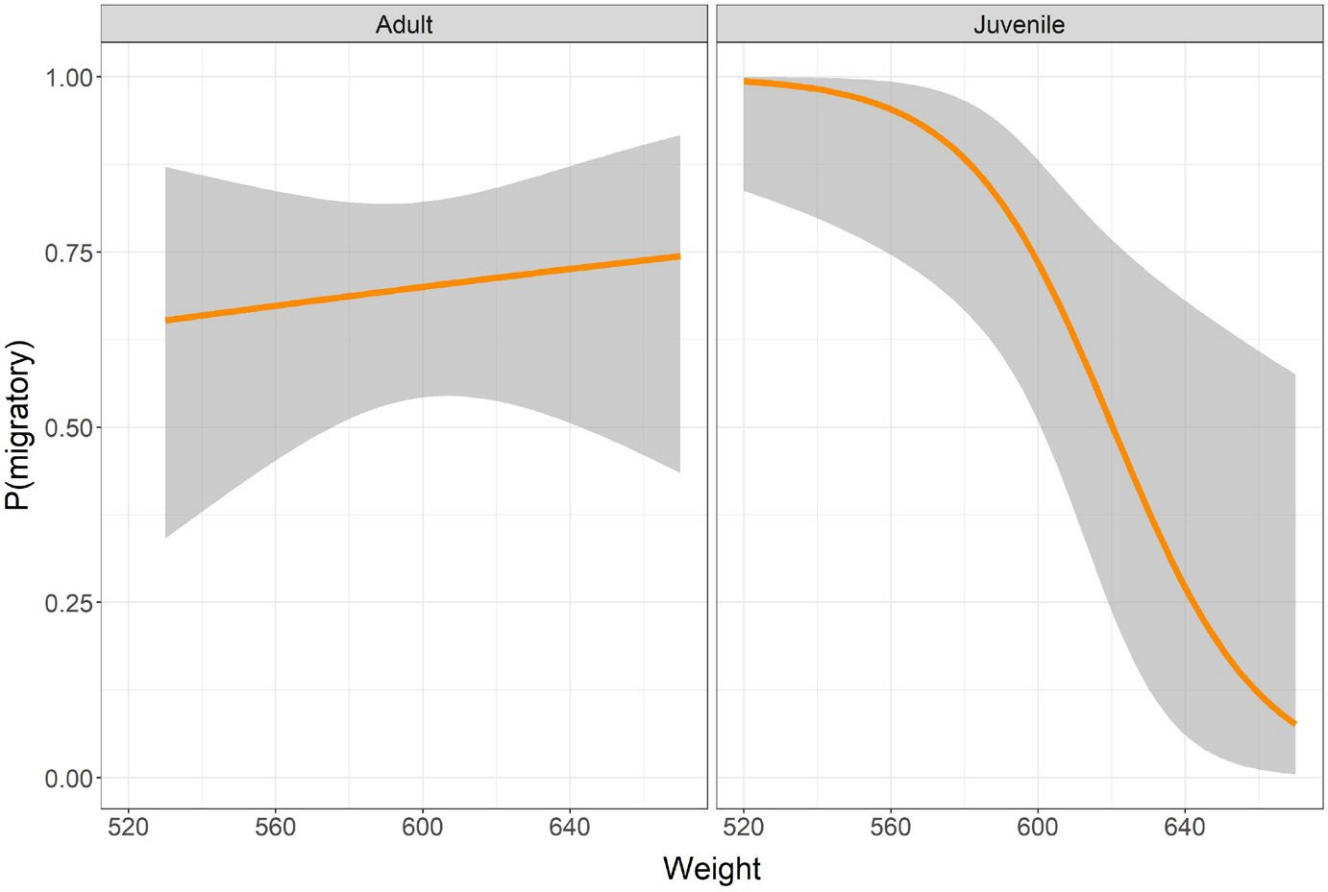
Estimated relationship (solid line) between body weight (g) and the probability of deciding to migrate in adult and juvenile female willow ptarmigan. The shaded ribbons represent 95% confidence interval. Only winter‐to‐summer transitions are included, and only first year of data for each bird

When modeling the decision to migrate or remain resident (including only the first spring movement for each individual female ptarmigan) as a function of age and body weight, we found strongest support for the full model including the age x weight interaction (Table 4, Appendix S1). This is in partial support of our prediction 1. A similar result was found when including all data (i.e., repeated observations for some birds, and both spring‐ and autumn movements; Appendix S1). The full model received substantially more support than the second‐ranked model (Table 4). For juveniles, the probability of migrating decreased with body weight (Figure 5), and thus the probability of remaining resident increased with weight. For adults, there was no apparent influence of body weight on the decision to migrate or remain resident. When modeling movement distance as a function of age and weight (including only the first spring movement for each individual female ptarmigan), we found no support for a difference between juveniles and adults (Table 5, Appendix S1), and the intercept‐only model had lowest AIC_c_. Similar inference was made when including all observations (i.e., repeated observations for some birds, and both spring and autumn movements; Appendix S1).

**TABLE 4:** Candidate models and model statistics for modeling migration strategy (migrate vs. remain resident) as a function of age (juvenile or adult) and body weight for female willow ptarmigan. Results from generalized linear models (GLMs) with binary response (1 = migrated, 0 = remained resident) and logit link function, assuming binomial error distribution. Only winter‐to‐summer migratory decisions are included

Response	Model	*K*	AIC_c_	ΔAIC_c_	AIC_c_Wt	CumWt
Migratory strategy	Weight + Age + Weight × Age	4	82.84	0.00	0.80	0.80
	Weight	2	87.50	4.66	0.08	0.88
	Intercept	1	87.78	4.94	0.07	0.95
	Age	2	89.60	6.75	0.03	0.97
	Weight + Age	3	89.61	6.76	0.03	1.00

**TABLE 5:** Candidate models and model statistics for modeling movement distance as a function of age (juvenile or adult) and body weight for female willow ptarmigan. Results from linear models (LMs) with continuous response assuming Gaussian error distribution. Only winter‐to‐summer transitions are included, and only first year of data for each bird

Response	Model	*K*	AIC_c_	ΔAIC_c_	AIC_c_Wt	CumWt
Distance	Intercept	2	298.58	0.00	0.48	0.48
	Weight	3	300.60	2.02	0.17	0.65
	Age	3	300.70	2.12	0.16	0.81
	Weight + Age + Weight × Age	5	301.16	2.58	0.13	0.94
	Weight + Age	4	302.82	4.24	0.06	1.00

### 3.2 Repeatability of migratory behavior

**FIGURE 6:**
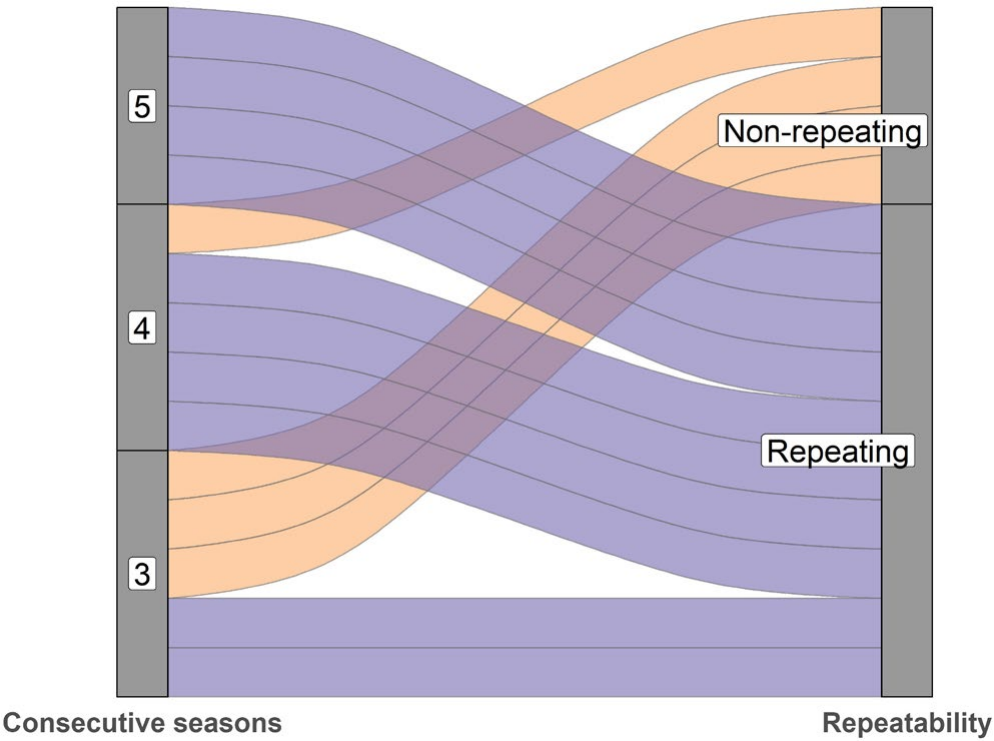
Repeatability of decision to migrate or remain resident between individuals. Purple bands = individuals with 100% repetition in migration decision between consecutive seasons. Orange bands = individuals that made different migration decisions in different seasons or years. Each band represents one individual

Repeatability of migratory behavior within individuals was very high (Figure 6), and repeatability within individuals increased each consecutive season. Among those individuals that changed migratory strategy, some were originally migratory, whereas others were originally resident. Agreement repeatability (based on the intercept‐only model) for movement distance revealed very high repeatability (*R* = 0.69, 95% CI = 0.36–0.85). Repeatability was equally high after accounting for potential age effects (i.e., adjusted repeatability) in movement distance (*R* = 0.71, 95% CI = 0.40–0.87).

### 3.3 Nesting success

In contrast to our third prediction, we did not find evidence that clutch size (Table 6, Appendix S1) or nest fate (Table 7, Appendix S1) varied as a function of migratory strategy, age, or weight. For both dependent variables, the ranking of models was identical (clutch size) or similar (nest fate) when including data beyond the first year after capture for each bird (Tables 6 and 7 vs. Appendix S1).

**TABLE 6:** Candidate models and model statistics for modeling number of laid eggs as a function of migratory strategy (migration vs. resident in wintering area), age (juvenile or adult), and body weight for female willow ptarmigan. Results from generalized linear models (GLMs) with count response and log link function, assuming generalized Poisson error distribution (see methods)

Response	Model	*K*	AIC_c_	ΔAIC_c_	AIC_c_Wt	CumWt
N eggs	Intercept	2	209.42	0.00	0.32	0.32
	Age	3	209.91	0.49	0.24	0.56
	Weight	3	211.33	1.91	0.12	0.68
	Migratory strategy	3	211.65	2.23	0.10	0.78
	Age + Weight	4	212.17	2.74	0.08	0.86
	Age + Migratory strategy	4	212.21	2.78	0.08	0.94
	Migratory strategy + Weight	4	213.62	4.20	0.04	0.98
	Migratory strategy + Age + Weight	5	214.53	5.11	0.02	1.00

**TABLE 7:** Candidate models and model statistics for modeling nest fate as a function of migratory strategy (migration vs. remain resident in wintering area), age (juvenile or adult), and body weight for female willow ptarmigan. Results from generalized linear models (GLMs) with binary response (1 = hatched, 0 = abandoned/predated) and logit link function, assuming binomial error distribution. Only data from first year after capture are used

Response	Model	*K*	AIC_c_	ΔAIC_c_	AIC_c_Wt	CumWt
Nest fate	Intercept	1	79.64	0.00	0.40	0.40
	Migratory strategy	2	81.56	1.93	0.15	0.56
	Age	2	81.73	2.10	0.14	0.70
	Weight	2	81.77	2.14	0.14	0.83
	Age + Migratory strategy	3	83.72	4.09	0.05	0.89
	Weight + Migratory strategy	3	83.80	4.16	0.05	0.94
	Age + Weight	3	83.94	4.30	0.05	0.98
	Migratory strategy + Weight + Age	4	86.04	6.40	0.02	1.00

## 4 DISCUSSION

We found that the willow ptarmigan population in the study area was partially migratory, and most (73%) of the individuals decided to carry out a seasonal migration from winter to summer areas rather than remaining resident. Similar migratory strategies have been reported from several other species of Galliformes, including spruce grouse *Falcipennis canadensis* (Herzog & Keppie, 1980) and blue grouse *Dendragapus obscurus* (Cade & Hoffman, 1993). Partly in line with our first prediction, we found that body weight related to the decision to migrate or to remain resident. This effect was only found among juvenile birds, where individuals with high body weight had a higher probability of remaining in the winter area. Among adult females, body weight did not appear to influence the decision to migrate or remain resident. In contrast with our second prediction, we found that migration decision was a fixed strategy once established, and individuals for which data on more than one seasonal migratory decision was available, showed a high degree of repeatability in migratory behavior. Finally, we found no support for our third prediction, as resident female willow ptarmigan had similar reproductive success to migrants.

### 4.1 Migration strategy in relation to age and body weight

One key finding of our study was that juvenile willow ptarmigan with small body sizes had a higher probability of migrating. The body size hypothesis posits that large body sizes will be advantageous to endure thermal variations and variation in food availability in harsh winter climates, and winter survival is generally high and stable in willow ptarmigan (Israelsen et al., 2020). Second, our data do not allow for an efficient test of this hypothesis because we only included birds with a shared winter area. Below, we discuss the likely importance of the dominance and the arrival time hypotheses for our results.

As posited by the dominance hypothesis, individuals with high body weight should have a competitive advantage to smaller individuals, forcing smaller individuals to migrate (Gauthreaux, 1982). For the dominance hypothesis to work, there must be an intraspecific competition for limited resources such as food or nest sites (Matthysen, 2005; Newton, 1998). Nesting sites close to the wintering grounds might be a limited resource (Gillis et al., 2008), and large dominant individuals might occupy the best breeding territories forcing juvenile ptarmigans to migrate to find a suitable breeding territory. This may be the case in the wintering areas where ptarmigan density is high during the winter months, and smaller (less dominant) individuals must migrate to find a suitable breeding territory in spring. Although two previous studies on dispersing juvenile willow ptarmigans in Scandinavia found no density dependence in dispersal rates (Brøseth et al., 2005; Hörnell‐Willebrand et al., 2014), intraspecific competition driven by positive density‐dependent factors might still be an important driver of partial migration in our study population.

Several studies have found support for the arrival time hypothesis as a driver of partial migration (Fudickar et al., 2013; Ketterson & Nolan, 1976; Lundblad & Conway, 2020), but lack of data on the when the females arrived in their breeding territories prevented us from testing this hypothesis explicitly. However, willow ptarmigans to some extent adjust the start of the breeding season to the timing of spring (Myrberget, 1986), hence, earlier spring leads to an early start to the breeding season. Resident ptarmigans may have an advantage in occupying high‐quality territories prior to migrating individuals, and this might be particularly true in years with mild winters and early spring.

Our finding that the decision to migrate or remain resident depended on body weight in juveniles but not in adults is only partly in line with the dominance hypothesis. However, if migration in juveniles is affected by density‐dependent factors, such as limitations in available territories, the dominance hypothesis may explain partial migration in juvenile ptarmigan.

### 4.2 Repeatability of migration strategy

Once established, migratory behavior seems to be a relatively fixed trait in our study population, and the repeatability in migration decisions within individuals was very high. Our findings are in line with several studies on breeding partial migratory populations, which have found migratory strategy to be fixed within individuals (Chambon et al., 2019; Gillis et al., 2008). For example, in a breeding partial migratory population of American crow *Corvus brachyhynchos* in USA, Townsend et al. (2018) found that migratory strategy was fixed within individuals, the proportion of migrants was 78% and with high breeding site fidelity. Interestingly, bird populations that breed sympatrically but winter allopatrically seem to have a higher degree of non‐fixed migration behavior (Dale et al., 2019; Hegemann et al., 2015; Lundblad & Conway, 2020).

A potential benefit of a fixed migratory strategy may be less exposure to unfamiliar habitat, and higher mortality rates that are associated by switching breeding sites between years (often referred to as breeding dispersal) have been reported (Bonte et al., 2011; Daniels & Walters, 2000; Greenwood & Harvey, 1982). Returning to the same breeding territory may also be beneficial due to familiarity with food resources and shelter from predators, which in turn leads to a more efficient use of resources (Greenwood & Harvey, 1982). This effect may be enhanced in individuals that remain resident all year, and according to Buchan et al. (2019) most studies on the consequences of partial migration reported higher mortality in migrants than in resident individuals. The high repeatability in migratory strategy within willow ptarmigans may be caused by resistance against moving to unfamiliar breeding wintering sites.

### 4.3 Reproductive success in relation to migration strategy

In contrast to our third prediction, we did not find any statistical support for higher reproductive success (measured as clutch size and nest fate) of resident birds. Our prediction was based on the “best of a bad job” hypothesis (Lundberg, 1987), positing that migration is a losing strategy that should lead to reduced fitness. Based on a multi‐taxa assessment, Buchan et al., 2019 reported that although most studies reported fitness differences between resident and migrants (73% of the studied populations reported higher fitness of residents, 22% reported higher fitness of migrants, and 5% reported equal fitness), fitness differences were most often caused by differences in survival. They argue that the reason for this finding can be that anthropogenic changes reduce the survival of migratory individuals. Our finding that migratory decisions seem to be relatively fixed once established appears to be in line with the finding that fitness does not differ between the strategies in our study population. However, there may be differences in survival between residents and migrants, and we suggest further investigations to be carried out to get a better understanding of the consequences of partial migration in the willow ptarmigan.

For fitness to be equal between the two migratory strategies, theoretical studies suggest that higher survival in migrants must offset the increased nesting success in residents (Chapman et al., 2011; Lundberg, 1987). Reduced risk of predation (Hebblewhite & Merrill, 2007; Skov et al., 2011), escape from harsh climatic conditions, and better forage are pointed at as important factors enhancing survival in migrants. Our results showed that a large proportion of the willow ptarmigan population carried out seasonal migrations, with little variation between years. If migratory strategy is genetically determined, the fitness trade‐off between migrating vs. resident strategies may be frequency dependent where the fitness payoff for each genotype increases or decreases with the genotype's frequency in the population (Heino et al., 1998; Lundberg, 1987). Negative frequency‐dependent selection rewards the strategy with lowest frequency in the population, i.e., selection is density dependent. The population may reach an equilibrium in an evolutionary stable state between migrants and residents where both strategies (genetic morphs) yield the same fitness. The frequencies of migrants and residents may stabilize at any ratio, and the small between‐year changes in the migrants:residents ratio in this willow ptarmigan population may indicate that it is in equilibrium. This may explain why we did not find any differences in reproductive success between the two strategies. If this is indeed the case, migrants are not making “the best of a bad job” where migration is the losing strategy in terms of both survival and reproductive success, and contradicts the findings of most empirical studies (Buchan et al., 2019; Chapman et al., 2011).

To conclude, we found that willow ptarmigans in central Norway were partially migratory, making them well suited for studies of the evolution of partial migration. The probability of remaining resident in the wintering area increased with increased body weight in juveniles, but not in adults. We found partial support for the dominance hypothesis for explaining partial migration, but cannot exclude the arrival time hypothesis as a potential driver of the observed pattern. The migratory decisions displayed at the juvenile stage appeared to become fixed throughout the individuals’ lifetime. We found no difference in average reproductive success between migratory strategies, which indicates that both strategies yield equal fitness unless there are differences in survival between the strategies.

## AUTHOR CONTRIBUTION

**Øyvind Arnekleiv:** Formal analysis (lead); Methodology (equal); Visualization (lead); Writing – original draft (lead). **Katrine Eldegard:** Conceptualization (supporting); Formal analysis (supporting); Methodology (supporting); Supervision (equal); Validation (supporting); Visualization (supporting); Writing – original draft (supporting); Writing – review & editing (equal). **Pål F. Moa:** Conceptualization (supporting); Data curation (supporting); Funding acquisition (supporting); Project administration (supporting); Supervision (supporting); Writing – original draft (supporting); Writing – review & editing (supporting). **Lasse F. Eriksen:** Conceptualization (supporting); Data curation (supporting); Formal analysis (supporting); Methodology (supporting); Supervision (supporting); Writing – review & editing (equal). **Erlend B. Birkeland Nilsen:** Conceptualization (equal); Data curation (supporting); Formal analysis (equal); Funding acquisition (lead); Methodology (equal); Project administration (lead); Supervision (lead); Validation (equal); Visualization (supporting); Writing – original draft (supporting); Writing – review & editing (lead).

## Supporting information

Appendix S1

## Data Availability

Data and R‐code (Arnekleiv et al., 2022) are available from a time‐stamped registered archive at Open Science Framework (DOI: https://doi.org/10.17605/OSF.IO/CY68W).
