# Supplementary material for: Drivers and consequences of partial migration in an alpine bird species
Source: Ecol Evol. 2022 Mar 10;12(3):e8690. doi: 10.1002/ece3.8690 (PMC8928885; doi:10.1002/ece3.8690)
Supplement: Supplementary file 1 — Appendix S1 [file ECE3-12-e8690-s001.docx]

# Appendix A

## Parameter estimates for the two best models from each analysis

**Table A1:** Two best models when modelling migration strategy as a function of age (juvenile or adult) and body weight. Results from generalized linear models (GLMs) with binary response (Y = 1 = migrated, Y = 0 = remained resident) and logit link function, assuming binomial error distribution. Only winter to summer transitions are included, and only first year of data for each bird.

| **Model 1: Migratory strategy ~ Weight × Age** | **Estimate** | **Std.Error** |
| --- | --- | --- |
|  |  |  |
| Intercept | 0.82 | ± 0.35 |
| Weight | 0.13 | ± 0.33 |
| Age(juv) | 0.61 | ± 0.35 |
| Weight × Age(juv) | -2.18 | ± 0.88 |
|  |  |  |
| **Model 2: Migratory strategy ~ Weight** |  |  |
|  |  |  |
| Intercept | 1.04 | ± 0.27 |
| Weight | -0.42 | ± 0.27 |

**Table A2:** Two best model when modelling log(distance moved) as a function of age (juvenile or adult) and body weight. Results from linear models assuming Gaussian error distribution. Only winter to summer transitions are included, and only first year of data for each bird.

| **Model 1: log(Distance) ~ Intercept** | **Estimate** | **Std.Error** |
| --- | --- | --- |
|  |  |  |
| Intercept | 1.22 | ± 0.21 |
|  |  |  |
| **Model 2: log(Distance) ~ Age** |  |  |
| Intercept | 1.22 | ± 0.22 |
| Weight | -0.08 | ± 0.22 |
|  |  |  |

**Table A3:** Two best models when modelling number of eggs laid as a function of age (juvenile or adult), body weight and migratory strategy. Results from generalized linear models (GLMs) with count response and log link function, assuming Conway-Maxwell Poisson error distribution. Only winter to summer transitions are included, and only first year of data for each bird.

| **Model 1: Number of eggs ~ Intercept** | **Estimate** | **Std.Error** |
| --- | --- | --- |
|  |  |  |
| Intercept | 2.26 | ± 0.026 |
|  |  |  |
| **Model 2: Number of eggs ~ Age** |  |  |
|  |  |  |
| Intercept | 2.30 | ± 0.03 |
| Age (juv) | -0.07 | ± 0.05 |

**Table A4:** Two best models when modelling nest fate as a function of age (juvenile or adult), body weight and migratory strategy. Results from generalized linear models (GLMs) with binary response (Y = 1 = hatched, Y = 0 = abandoned/predated) and logit link function, assuming binomial error distribution. Only winter to summer transitions are included, and only first year of data for each bird.

| **Model 1: Nest fate ~ Intercept** | **Estimate** | **Std.Error** |
| --- | --- | --- |
|  |  |  |
| Intercept | 0.07 | ± 0.27 |
|  |  |  |
| **Model 2: Nest fate ~ Age** |  |  |
|  |  |  |
| Intercept | 0.13 | ± 0.35 |
| Age (juv) | -0.13 | ± 0.54 |

## AICc-tables for mixed effects models based on pooled data from all transitions

**Table A5:** Candidate models and model statistics for modelling migration strategy as a function of age (juvenile or adult) and body weight for female willow ptarmigan. Results from generalized linear mixed models (GLMMs) with binary response (Y = 1 = migrated, Y = 0 = remained resident) and logit link function, assuming binomial error distribution. Individual identity was included as random effect to account for repeated observations of the same birds.

| Response | Model | K | AIC_c_ | ΔAIC_c_ | AIC_c_Wt | CumWt | | |
| --- | --- | --- | --- | --- | --- | --- | --- | --- |
| Migratory | Weight + Age + Weight × Age | 5 | 91.77 | 0.00 | 0.81 | | 0.81 |  |
| strategy | Intercept | 2 | 96.23 | 4.47 | 0.09 | | 0.90 |  |
|  | Weight | 3 | 97.58 | 5.81 | 0.04 | | 0.94 |  |
|  | Age | 3 | 98.05 | 6.28 | 0.04 | | 0.98 |  |
|  | Weight + Age | 4 | 99.00 | 7.24 | 0.02 | | 1.00 |  |

**Table A6**: Candidate models and model statistics for modelling movement distance as a function of age (juvenile or adult) and body weight for female willow ptarmigan. Results from linear mixed models (LMMs) with continuous response assuming Gaussian error distribution. Individual identity was included as random effect to account for repeated observations of the same birds.

| Response | Model | K | AICc | ΔAIC_c_ | | AIC_c_Wt | | CumWt |
| --- | --- | --- | --- | --- | --- | --- | --- | --- |
| Distance | Intercept | 3 | 390.93 | 0.00 | 0.43 | | 0.43 | |
|  | Age | 4 | 392.49 | 1.56 | 0.20 | | 0.63 | |
|  | Weight | 4 | 392.77 | 1.84 | 0.17 | | 0.80 | |
|  | Weight + Age + Weight ×Age | 6 | 393.58 | 2.65 | 0.12 | | 0.92 | |
|  | Weight + Age | 5 | 394.28 | 3.35 | 0.08 | | 1.00 | |

**Table A7**: Candidate models and model statistics for modelling number of laid eggs as a function of migratory strategy, age (juvenile or adult) and body weight for female willow ptarmigan. Results from generalized linear mixed models (GLMMs) with count response and log link function, assuming generalized Poisson error distribution (see methods). Individual identity was included as random effect to account for repeated observations of the same birds.

| Response | Model | K | AIC_c_ | ΔAIC_c_ | AIC_c_Wt | CumWt |
| --- | --- | --- | --- | --- | --- | --- |
| N eggs | Intercept | 3 | 239.22 | 0.00 | 0.33 | 0.33 |
|  | Age | 4 | 240.18 | 0.96 | 0.20 | 0.53 |
|  | Weight | 4 | 240.98 | 1.76 | 0.14 | 0.67 |
|  | Migratory strategy | 4 | 241.36 | 2.14 | 0.11 | 0.78 |
|  | Age + Weight | 5 | 242.26 | 3.04 | 0.07 | 0.86 |
|  | Age + Migratory strategy | 5 | 242.32 | 3.10 | 0.07 | 0.93 |
|  | Migratory strategy + Weight | 5 | 243.03 | 3.81 | 0.05 | 0.97 |
|  | Migratory strategy + Age + Weight | 6 | 244.35 | 5.13 | 0.03 | 1.00 |

**Table A8:** Candidate models and model statistics for modelling nest fate as a function of migratory strategy, age (juvenile or adult) and body weight for female willow ptarmigan. Results from generalized linear mixed models (GLMMs) with binary response (Y = 1 = hatched, Y = 0 = abandoned/predated) and logit link function, assuming binomial error distribution. Individual identity was included as random effect to account for repeated observations of the same birds.

| Response | Model | K | AIC_c_ | ΔAIC_c_ | AIC_c_Wt | CumWt |
| --- | --- | --- | --- | --- | --- | --- |
| Nest | Intercept | 2 | 88.78 | 0.00 | 0.42 | 0.42 |
| fate | Age | 3 | 90.95 | 2.17 | 0.14 | 0.57 |
|  | Migratory strategy | 3 | 90.98 | 2.20 | 0.14 | 0.71 |
|  | Weight | 3 | 90.99 | 2.21 | 0.14 | 0.85 |
|  | Age + Migratory strategy | 4 | 93.23 | 4.45 | 0.05 | 0.90 |
|  | Age + Weight | 4 | 93.24 | 4.46 | 0.05 | 0.94 |
|  | Migratory strategy + Weight | 4 | 93.27 | 4.49 | 0.04 | 0.99 |
|  | Migratory strategy +Age + Weight | 5 | 95.60 | 6.82 | 0.01 | 1.00 |
